# P17 Challenges of managing MDR infections in severe trauma ICU patients

**DOI:** 10.1093/jacamr/dlag102.023

**Published:** 2026-06-26

**Authors:** Nesrine Hagui, Mariem Gargouri

**Affiliations:** Center of Trauma and Major Burns, Fattouma Bourguiba Hospital, Monastir, Tunisia; Center of Trauma and Major Burns, Fattouma Bourguiba Hospital, Monastir, Tunisia

## Abstract

**Background:**

Trauma ICU patients at the Centre for Trauma and Major Burns (CTMB) are at high risk of nosocomial infections, often caused by MDR bacteria. This retrospective study aims to describe patient characteristics, bacterial ecology, and the impact of therapeutic strategies on morbidity and mortality.

**Methods:**

The study included 15 patients admitted to the ICU between January 1 and December 31, 2025. Collected data included age, sex, isolated pathogens, administered antibiotic therapy, length of stay, and clinical outcomes. Analysis focused on correlations between bacterial resistance, treatment strategies, and morbidity/mortality.

**Results:**

The cohort was predominantly young males (73%), with a mean age of 43.6 years. Overall mortality was 33%, mainly affecting older or frail patients. *Acinetobacter baumannii* was the most frequently isolated MDR organism, often associated with co-infections by *Pseudomonas*, *Klebsiella pneumoniae*, or *Candida*. Management required rapid escalation to last-resort antibiotics, mainly colistin and tigecycline, often in combination for MDR coverage. Early pathogen–treatment adequacy (<48 h) significantly improved survival. Exposure to more than four lines of therapy was associated with 60% mortality. Prolonged treatments (>10 days) increased the risk of candidiasis and extended the average ICU stay to 16.5 days.

**Conclusions:**

Patient survival depended on both early, appropriate therapy and baseline patient frailty. High selective bacterial pressure and polymicrobial infections complicated management, highlighting the need for strict hygiene, antimicrobial stewardship, and active surveillance of MDR carriers. At the CTMB, controlling infection risk remains a major challenge. MDR bacteria, dominated by *A. baumannii*, necessitate reserve antibiotics and underscore the importance of strengthened prevention, rapid optimized therapy, and systematic surveillance to improve outcomes in severely traumatized patients.

